# Physiological Roles of ArcA, Crp, and EtrA and Their Interactive Control on Aerobic and Anaerobic Respiration in *Shewanella oneidensis*

**DOI:** 10.1371/journal.pone.0015295

**Published:** 2010-12-28

**Authors:** Haichun Gao, Xiaohu Wang, Zamin K. Yang, Jingrong Chen, Yili Liang, Haijiang Chen, Timothy Palzkill, Jizhong Zhou

**Affiliations:** 1 Institute of Microbiology and College of Life Sciences, Zhejiang University, Hangzhou, Zhejiang, China; 2 Institute for Environmental Genomics and Department of Botany and Microbiology, University of Oklahoma, Norman, Oklahoma, United States of America; 3 Department of Pharmacology & Department of Molecular Virology and Microbiology, Baylor College of Medicine, Houston, Texas, United States of America; 4 Environmental Sciences Division, Oak Ridge National Laboratory, Oak Ridge, Tennessee, United States of America; Vrije Universiteit Brussel, Belgium

## Abstract

In the genome of *Shewanella oneidensis*, genes encoding the global regulators ArcA, Crp, and EtrA have been identified. All these proteins deviate from their counterparts in *E. coli* significantly in terms of functionality and regulon. It is worth investigating the involvement and relationship of these global regulators in aerobic and anaerobic respiration in *S. oneidensis*. In this study, the impact of the transcriptional factors ArcA, Crp, and EtrA on aerobic and anaerobic respiration in *S. oneidensis* were assessed. While all these proteins appeared to be functional *in vivo*, the importance of individual proteins in these two major biological processes differed. The ArcA transcriptional factor was critical in aerobic respiration while the Crp protein was indispensible in anaerobic respiration. Using a newly developed reporter system, it was found that expression of *arcA* and *etrA* was not influenced by growth conditions but transcription of *crp* was induced by removal of oxygen. An analysis of the impact of each protein on transcription of the others revealed that Crp expression was independent of the other factors whereas ArcA repressed both *etrA* and its own transcription while EtrA also repressed *arcA* transcription. Transcriptional levels of *arcA* in the wild type, *crp*, and *etrA* strains under either aerobic or anaerobic conditions were further validated by quantitative immunoblotting with a polyclonal antibody against ArcA. This extensive survey demonstrated that all these three global regulators are functional in *S. oneidensis*. In addition, the reporter system constructed in this study will facilitate *in vivo* transcriptional analysis of targeted promoters.

## Introduction

Depending on the availability of electron donors and acceptors, facultative anaerobes such as *Escherichia coli* adopt three different metabolic modes: aerobic respiration, anaerobic respiration, and fermentation [1]–[2]. Aerobic respiration with oxygen as the terminal electron acceptor (EA) is the most productive mode due to complete oxidation of a growth substrate while anaerobic respiration can only oxidize a substrate partially with alternative electron acceptors, such as nitrate. Fermentation is the least productive process because only substrate level phosphorylation occurs. As a result, aerobic respiration is preferred over anaerobic respiration, which in turn is preferred over fermentation [3].

Since molecular oxygen confers enormous energetic benefits, it is not surprising that changes in its availability lead to substantial changes in *E. coli* physiology. Several sensing systems are employed in *E. coli* to monitor environmental oxygen and cellular redox state. The switch between aerobic and anaerobic metabolism is controlled primarily by the Fnr (fumarate nitrate regulator) transcription factor and the Arc (aerobic respiration control) two-component regulatory system [3]. Fnr, synthesized under both aerobic and anaerobic conditions, is able to sense oxygen directly using its [4Fe-4S]^2+^ cluster whereas the Arc system senses oxygen indirectly [4]. After sensing changes in the redox state of the quinone pool elicited by oxygen limitation, ArcB, the sensor kinase of the system, autophosphorylates and then transphosphorylates the response regulator ArcA [5]–[6].

*Shewanella oneidensis* MR-1 is a facultative Gram-negative anaerobe with remarkable anaerobic respiration abilities that allow the use of a diverse array of terminal EAs [7]. Although little is known about how this bacterium adopts different metabolic modes in response to the availability of oxygen, surprising observations have been made. First, *S. oneidensis* does not ferment, although genes encoding many enzymes of mixed acid fermentation are present in the genome [8]. Second, EtrA (electron transport regulator A), the analog of *E. coli* Fnr, appears to have no significant role in mediating gene expression in response to oxygen availability [9]–[10]. Third, it is evident that Crp (cyclic-AMP receptor protein) is crucial in anaerobic respiration because *crp* mutants are defective in utilizing several EAs, including Fe^3+^, Mn^4+^, nitrate, fumarate, and dimethyl sulfoxide (DMSO) [11]. Fourth, *S. oneidensis* possesses an atypical Arc system in which function of ArcB is fulfilled by two proteins ArcS and HptA [12]. Unlike the Arc system in *E. coli*, this atypical system appears to be important in aerobic respiration and is not involved in regulation of TCA genes [13].

In the present study, we report results from experiments designed to reveal the involvement of three global regulators (ArcA, Crp, and EtrA) in aerobic and anaerobic respiration in *S. oneidensis*. For this purpose, seven strains, each of which has at least one of these three genes deleted, were constructed and characterized. In addition, a LacZ reporter system was developed for Gram-negative bacteria lacking a *lacZ* analog to investigate the interactive control among these regulators at the transcriptional level. The results were validated by quantitative western blotting with antibodies against *S. oneidensis* ArcA proteins. The results indicate that these transcription factor proteins are functional *in vivo* and respond with unique characteristics to the availability of oxygen and other transcription proteins.

## Results

### Generation of *arcA*, *crp*, and *etrA* single, double, and triple deletion mutants

Individual chromosomal mutants of *arcA*, *crp*, and *etrA* have been generated and characterized [9], [11], [13]–[14]. While an *arcA* mutant, designated as JZ3988K (*ΔarcA*), was constructed and validated as a deletion, the *crp* and *etrA* constructs reported previously were insertion mutants. Unfortunately, the first *etrA* mutant was later invalidated [9], [14]. As a result, concerns about insertional mutagenesis in *S. oneidensis* have been raised, especially with MR-1R as the parental strain. MR-1R carries a mutation resulting in a rifampin resistance phenotype, which causes a decreased menaquinone level [15]. This complicates physiological observations on growth under anaerobic conditions because the menaquinone pool is the electron source for terminal reductases in anaerobic respiration.

Given their importance in respiration of *E. coli* and *S. oneidensis*, the creation and validation of a collection of mutants in which one or more of the *arcA*, *crp*, and *etrA* genes is (are) deleted would be indispensable for a quantitative characterization of global transcriptional regulation. To this end, the *crp* and *etrA* single deletion mutants were constructed, named JZ0624 (*Δcrp*) and JZ2356 (*ΔetrA*) respectively. With one of these single mutants as the parental strain, double mutants JZ3988K-0624 (*ΔarcAΔcrp*), JZ3988K-2356 (*ΔarcAΔetrA*), and JZ0624-2356 (*ΔcrpΔetrA*), and the triple mutant JZ3988K-0624-2356 (*ΔarcAΔcrpΔetrA*) were generated by simply repeating the mutagenesis procedure. The deletion(s) in all of these strains as listed in Table 1 were verified by PCR and DNA sequencing.

**Table 1:** Strains and plasmids used in this study.

Strain or plasmid	Description	Reference or source
*E. coli* strain		
BL21	F^−^ *omp*T *hsd*S_B_(r_B_^−^m_B_^−^) *gal dcm* (DE3)	GE Healthcare
WM3064	Donor strain for conjugation; Δ*dapA*	[16]
*S. oneidensis* strains		
MR-1	Wild-type	ATCC 700550
JZ3988K	*arcA* deletion mutant derived from MR-1; Δ*arcA*	[13]
JZ0624	*crp* deletion mutant derived from MR-1; Δ*crp*	This study
JZ2356	*etrA* deletion mutant derived from MR-1; Δ*etrA*	This study
JZ3988-0624	*arcA* and *crp* double deletion mutant derived from MR-1; Δ*arcA*Δ*crp*	This study
JZ3988-2356	*arcA* and *etrA* double deletion mutant derived from MR-1; Δ*arcA*Δ*etrA*	This study
JZ0624-2356	*crp* and *etrA* double deletion mutant derived from MR-1; Δ*crp*Δ*etrA*	This study
JZ3988-0624-2356	*arcA*, *crp*, and *etrA* triple deletion mutant derived from MR-1; Δ*arcA*Δ*crp*Δ*etrA*	This study
Plasmids		
pDS3.0	Ap^r^, Gm^r^, derivative from suicide vector pCVD442	[17]
pDS-ARCAK	pDS3.1 containing the PCR fragment for deleting *arcA*	[13]
pDS-CRP	pDS3.1 containing the PCR fragment for deleting *crp*	This study
pDS-ETRA	pDS3.1 containing the PCR fragment for deleting *etrA*	This study
pCM62	Base plasmid for constructing a reporter system	[18]
pBlueSTAR-1	Full length of *lacZ* gene template	Novagen
pTP325	pCM62 with a full length *lacZ* gene	This study
pTP327	pTP325 without P*lac* promoter	This study
pTP327-ARCAp	pTP327 containing the *S. oneidensis arcA* promoter	This study
pTP327-CRPp	pTP327 containing the *S. oneidensis crp* promoter	This study
pTP327-ETRAp	pTP327 containing the *S. oneidensis etrA* promoter	This study

### Heme *c* levels in mutants

**Figure 1:**
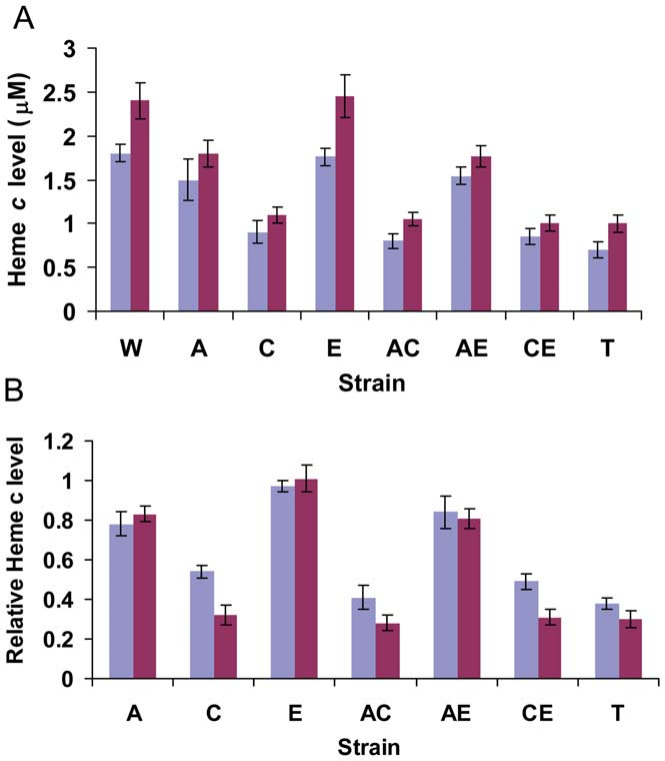
Heme *c* levels in the *S. oneidensis* wild type and *arcA*, *crp*, *etrA* deletion mutant strains. Cells at an OD_600_ of 0.6 under aerobic conditions and an OD_600_ of 0.25 under anaerobic conditions were collected for the heme *c* assay (see text for details). Three independently collected samples were assayed and the average level was presented. Error bars represent the standard deviations of the data. (A) The absolute levels of Heme *c* (µM per g of proteins) in the *S. oneidensis* wild type and *arcA*, *crp*, *etrA* deletion mutant strains under either aerobic (blue) or anaerobic (red) growth conditions were recorded (B) The relative heme *c* levels in deletion mutant strains under either aerobic (blue) or anaerobic (red) growth conditions. Values are relative to that in wild type MR-1. In all panels, W, MR-1; A, Δ*arcA*; C, Δ*crp*; E, Δ*etrA*; AC, Δ*arcA*Δ*crp*; AE, Δ*arcA*Δ*etrA*; CE, Δ*crp*Δ*etrA*; T, Δ*arcA*Δ*crp*Δ*etrA*.

It has been reported that *S. oneidensis* contains 42 genes for predicted *c*-type cytochromes [19] and the latest analysis suggests that the genome possesses 41 genes encoding intact *c*-type cytochrome proteins [20]. As the main components of respiratory electron transport chains, it is conceivable that cellular levels of heme *c* (components of *c*-type cytochromes) could be affected by mutations in the genes encoding the major transcriptional regulatory factors controlling bacterial aerobic and anaerobic respiration. To assess impacts of ArcA, EtrA and Crp on cellular levels of *c*-type cytochromes, the wild-type and mutant cells grown to an OD_600_ of 0.6 under aerobic conditions and to an OD_600_ of 0.25 under anaerobic conditions were collected and cellular levels of heme *c* in these samples were examined and results were shown in Fig. 1A. The wild type cells grown under aerobic and anaerobic conditions contained approximately 1.8 µM and 2.4 µM concentration of heme *c* per g of protein, respectively. Levels of cellular heme *c* in mutant strains varied significantly, with an average of 0.9/1.05 (aerobic/anaerobic, the same below) in any mutant devoid of *crp*, an average of 1.5/1.8 in *ΔarcA* and *ΔarcAΔetrA*, and an average of 1.8/2.4 in *ΔetrA*. To facilitate the comparison of mutation effects on the cellular heme *c* levels, relative heme *c* levels of in each mutant were presented as the ratios of the absolute amount of heme *c* of each mutant *vs.* MR-1 under the same growth conditions (Fig. 1B). All of these results demonstrated that 1) the amount of heme *c* in each strain was higher when grown under anaerobic conditions; 2) relative heme *c* level in each mutant appeared to be similar irrespective of growth conditions although phenotypes of certain mutants were only observed under either aerobic or anaerobic condition; 3) mutants in which *crp* was deleted exhibited significantly lower levels of heme *c* than the wild type, whereas mutations in *arcA* showed some mild negative influence on heme *c* synthesis and *etrA* did not affect heme *c* levels.

### Physiological analysis of the mutants

The single, double and triple deletion mutant strains were then subjected to physiological characterization as performed previously for the *arcA* deletion mutant JZ3988K [13]. The respiratory conditions tested in the characterization included: (i) aerobiosis, and (ii) anaerobiosis with a variety of electron acceptors (EA) including fumarate (20 mM), nitrate (3 mM), dimethyl sulfoxide (DMSO 20 mM), trimethylamine *N*-oxide (TMAO 20 mM), thiosulfate (3 mM), MnO_2_ (5 mM), ferric citrate (10 mM), and FeO(OH) (10 mM).

**Figure 2:**
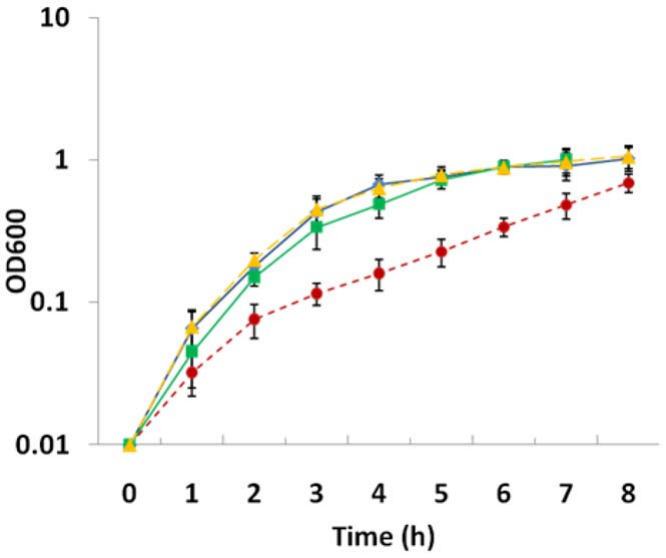
Aerobic growth of *S. oneidensis* MR-1 and mutant strains. All strains were grown in the defined medium under aerobic conditions and growth was monitored at OD_600_. For clarification, only wild-type (Blue Diamond) and single mutants Δ*etrA* (Yellow Triangle), Δ*crp* (Green Square), and Δ*arcA* (Brown Circle) were presented in the figure. Strains with indistinguishable phenotypes: MR-1  =  Δ*etrA*; Δ*crp  = * Δ*crp*Δ*etrA*; Δ*arcA  = *Δ*arcA*Δ*crp  = * Δ*arcA*Δ*etrA  = * Δ*arcA*Δ*crp*Δ*etrA*.

In order to precisely assign observed phenotypes to individual mutations, physiological differences of three single mutants under aerobic and anaerobic conditions were examined. The results presented in Fig. 2 indicate that a mutation in *arcA* results in a substantially slower growth rate of cells under aerobic conditions while the growth rate of strains carrying mutation in either *crp* or *etrA* was identical to that of wild type MR-1, consistent with previous reports [1], [11], [13]–[14]. Dissolved oxygen in all cultures was monitored as done previously with the *ΔarcA* strain and the same result was obtained (data not shown) [13]. In all cases, DO decreased quickly at the early stage, reached the lowest point at the mid-log phase and remained at the level until the late stationary phase, suggesting that DO is a function of the cell density but not related to the genotype of individual strains. Not surprisingly, the double mutants (*ΔarcAΔcrp* and *ΔarcAΔetrA*) and the triple mutant (*ΔarcAΔcrpΔetrA*) shared a similar phenotype of slower aerobic growth with the *ΔarcA* strain while the double mutant *ΔcrpΔetrA* was not defective in growth rate (data not shown). These results strongly suggest that *arcA* but not *crp* or *etrA* plays a substantial role in the bacterial aerobiosis.

Under anaerobic growth conditions, the utilization of EAs by the mutants was investigated by following methods. The ability to utilize fumarate, nitrate, DMSO, TMAO, or thiosulfate was assessed by measuring culture turbidity. Utilization of MnO_2_, ferric citrate, and cobalt(III)-EDTA was assayed by the color change of cultures and validated by chemical analysis as described in Methods. The results clearly revealed that Crp plays an important role in *S. oneidensis* anaerobic respiration (Table 2). Among the tested EAs, only TMAO allows cells of the *Δcrp* strain to grow, as reported previously (Table 2) [11]). In contrast, the *ΔetrA* strain did not show distinguishable defects in growth with any of the tested EAs compared to the parental strain MR-1 (Table 2). It was reported previously that an insertional *etrA* mutant differed noticeably in utilization of fumarate and nitrate from MR-1 [14]. For verification, an IC (Ion Chromatography) analysis on nitrate and nitrite levels in cultures of the *ΔetrA* strain and MR-1 was performed. No statistically significant difference in growth characteristics was detected among the cultures (Table 2). Finally, the *ΔarcA* strain was defective in utilization of DMSO only, as reported elsewhere [13], [21].

**Table 2:** Growth differences of mutants compared to the wild type under anaerobic conditions.

Strain	Electron Acceptor (EA)^a^							
	Fumarate	DMSO	TMAO	Nitrate	FeO(OH)	Thiosulfate	Fe-Citrate	MnO_2_
*ΔarcA*	N^b^	D^c^	N	N	N	N	N	N
*Δcrp*	D	D	N	D	D	D	D	D
*ΔetrA*	N	N	N	N	N	N	N	N
*ΔarcAΔcrp*	D	D	N	D	D	D	D	D
*ΔarcAΔetrA*	N	D	N	N	N	N	N	N
*ΔcrpΔetrA*	D	D	N	D	D	D	D	D
Triple	D	D	N	D	D	D	D	D

a Used in defined medium supplemented with 20 mM lactate, concentrations of EAs refer to the text.

b Growth on or reduction of the electron acceptor used is normal (N, similar to the wild type)

c Growth on or reduction of the electron acceptor used is defective (D).

On the assumption based on the above experiments that EtrA does not play significant role in *S. oneidensis* anaerobic respiration, it is likely that the phenotypes of the double and triple mutants in this process result from the loss of *crp* or *arcA*. Experimental data on these mutants supported the assumption. All these mutants except the *ΔarcAΔetrA* strain were unable to reduce any of the EAs but TMAO. The *ΔarcAΔetrA* strain, as expected, was only deficient in DMSO reduction (Table 2).

### Phenotypic microarray analysis of the mutants

The canonical Crp as in *E. coli* is the primary sensor and transcriptional regulator of carbon metabolism [22]. Although function shift of *S. oneidensis* ArcA, Crp, and EtrA appears to be substantial, an examination of their canonical roles is much needed. To this end, the ability of the wild-type and all mutant strains to metabolize 190 different carbon sources under aerobic conditions was tested using PM1 phenotype microarrays from Biolog (www.biolog.com/pdf/PM1-PM10.pdf). Wild type MR-1 displayed positive reaction with 15 carbon sources presented in Table 3. While both the *ΔetrA* and *ΔcrpΔetrA* strains were able to utilize all these 15 carbon sources, strains carrying a deletion in *arcA* were metabolically more restricted. It is also evident that the *ΔarcA* single mutant and the *ΔarcAΔetrA* double mutant were virtually the same in their ability to metabolize these carbon sources. The *Δcrp* strain, however, appeared to be able to rescue defects of the *ΔarcA* strain on some carbon sources, such as glycyl-L-glutamic acid, tween 40, and tween 80 as revealed by the *ΔarcAΔcrp* and triple mutants. These findings suggest that ArcA plays a more important role in carbon metabolism than Crp and EtrA while the role of EtrA appears negligible.

**Table 3:** Carbon utilization of MR-1 and mutation strains by phenotypic microarray analysis^a^.

Metabolite	*ΔarcA*	*Δcrp*	*ΔetrA*	*ΔarcAΔcrp*	*ΔarcAΔetrA*	*ΔcrpΔetrA*	Triple
Glycyl-L-Aspartic Acid	N	0.85±0.13	1.13±0.17	N	N	2.63±0.27	N
Glycyl-L-glutamic Acid	N	1.93±0.20	1.26±0.09	0.94±0.13	N	2.12±0.31	1.29±0.08
N-Acetyl-D-Glucosamine	0.70±0.09	2.74±0.27	1.71±0.21	1.78±0.17	0.58±0.07	2.24±0.33	2.27±0.29
Tween 20	0.49±0.07	1.55±0.12	0.94±0.08	0.54±0.04	0.60±0.03	1.18±0.12	0.72±0.12
Tween 40	N	2.16±0.11	1.28±0.08	0.71±0.07	N	1.47±0.14	0.70±0.09
Tween 80	N	1.92±0.21	1.13±0.11	0.76±0.08	N	1.93±0.17	1.00±0.07
α-Keto-Butyric Acid	N	1.89±0.31	2.36±0.34	1.02±0.15	N	2.33±0.16	N
Pyruvic Acid	N	N	1.10±0.12	N	N	1.13±0.15	N
L-Lactic Acid	0.50±0.07	1.84±0.19	1.98±0.12	N	0.46±0.04	1.36±0.10	N
Methyl Pyruvate	0.92±0.06	2.13±0.04	2.16±0.06	0.64±0.07	1.06±0.10	1.44±0.17	1.03±0.06
2-Deoxy Adenosine	N	N	1.90±0.01	N	N	1.44±0.09	N
Uridine	0.81±0.11	1.41±0.15	2.55±0.09	0.99±0.08	1.02±0.09	2.47±0.35	1.83±0.10
Adenosine	0.81±0.05	1.79±0.07	1.92±0.11	1.24±0.09	1.09±0.06	1.58±0.16	1.58±0.13
Inosine	1.06±0.07	2.06±0.30	1.99±0.21	1.42±0.10	1.58±0.16	1.69±0.11	1.70±0.16
Gelatin	N	2.18±0.12	1.99±0.17	N	N	1.64±0.18	N

a Relative values were presented as signal reading of each mutant/signal reading of the wild-type strain. N represents negative results from signal reading of certain mutant with specific metabolite.

### Development and validation of a *lacZ* reporter system in *S. oneidensis*

In *E. coli*, ArcA, Crp and Fnr(EtrA) are important components of transcriptional regulatory networks and inter-regulation mechanisms among the transcription factors have been reported [23]. To explore whether such mechanisms are present in *S. oneidensis*, a β-galactosidase reporter assay was established for studying *in vivo* transcriptional regulation in *S. oneidensis*. A plasmid, pCM62, suitable for developing an *E. coli* LacZ reporter system has been widely used in a variety of Gram-negative bacteria including *S. oneidensis* [18], [24]. While the plasmid has been exploited for many different purposes, it cannot be directly applied to *S. oneidensis* as an *E. coli* LacZ reporter system for following two reasons. First, the *S. oneidensis* genome lacks a *lacZ* homolog gene, thus it is not able to provide *lacZβγ* to complement the *lacZα* peptide encoded within pCM62 to form a functional β-galactosidase unit. Second, the P*lac* promoter in front of *lacZα* within pCM62 is very likely to interfere with the *lacZ* expression from an inserted *S. oneidensis* promoter subjected to examination. It may be particularly important for this study because the P*lac* promoter has been shown to be regulated by Crp in *E. coli* [25].

**Figure 3:**
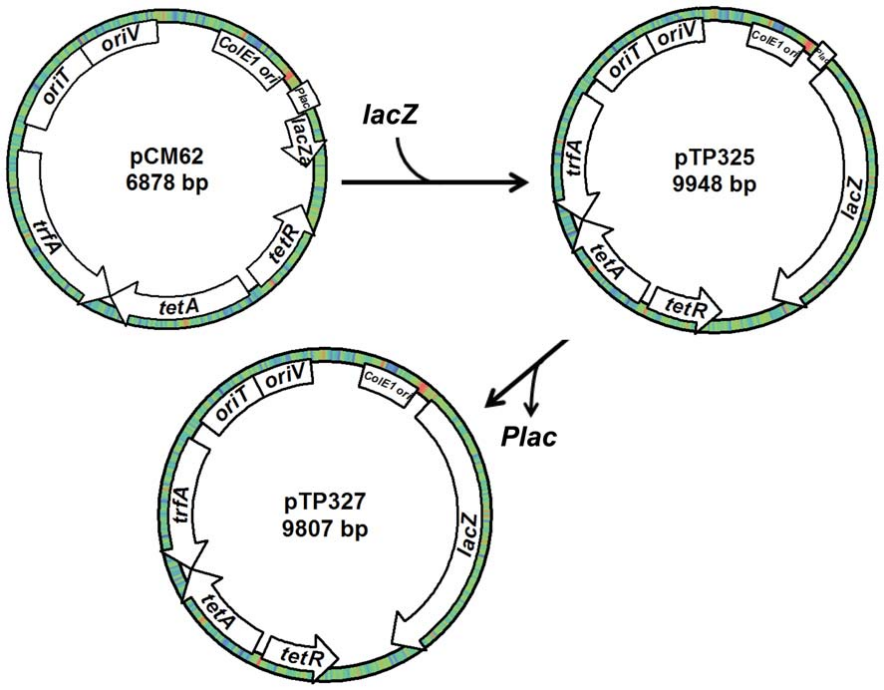
Construction of a *lacZ* reporter system. The full-length *lacZ* gene obtained by PCR was used to replace *lacZα* on pCM62. The P*lac* promoter was then removed from the resultant plasmid pTP325, resulting in the final plasmid pTP327.

To develop an *E. coli* LacZ reporter system for *S. oneidensis*, the *lacZα* gene of pCM62 was replaced with a full length *E. coli lacZ* gene generated by PCR with the template and primers listed in Table S1 in Supporting Information (Fig. 3). The resulting plasmid, pTP325, was further modified by substituting the P*lac* promoter located in front of the full length *E. coli lacZ* gene with a synthetic oligo (listed in Table S1), resulting in the promoterless pTP327. To test utility of pTP327 as a promoter-probe vector, the *arcA*, *crp*, or *etrA* promoters of *S. oneidensis* were transcriptionally fused to *lacZ* within pTP327, resulting in pTP327-ArcA, pTP327-Crp, and pTP327-EtrA, respectively. These plasmids, along with pCM62, pTP325, and pTP327, were individually introduced into *S. oneidensis* MR-1 for validation. The β-galactosidase activities in cells grown under aerobic conditions were measured as presented in Table 4. While similar reporter activities were obtained with pCM62 and pTP325 in *E. coli* under the same conditions (presence/absence of 0.5 mM IPTG), reporter activities of these plasmids in *S. oneidensis* differed significantly. Expectedly, pCM62 failed to produce β-galactosidase activity due to the lack of full-sized LacZ and pTP325 expressed *lacZ* constitutively because of the lack of LacI in the bacterium. This confers an advantage of blue-white screening to pTP325 over pCM62 as an expression vector in *S. oneidensis* and other Gram-negative bacteria without a *lacZ* homolog gene in their genome. A higher background β-galactosidase activity from pTP327 in *E. coli* than *S. oneidensis* was likely due to the fact that *E. coli* hosted a higher copy number of the plasmid because of its ColE1 *ori*. Other origins, which allow *S. oneidensis* to maintain the plasmid, restricted a high copy number in the microorganism [18].

**Table 4:** β-galactosidase activity present in *S. oneidensis* and *E. coli* cell extracts.

Plasmid	Features	β-galactosidase Activity (nmol min^−1^(mg protein)^−1^)	
		In *E. coli*	In *S. oneidensis*
pCM62	P*lac* + *lacZ*	42±7/2760±130^a^	1.7±0.4/2.1±0.5^a^
pTP325	P*lac* + *lacZ*	39±8/2840±125^a^	424±28/450±37^a^
pTP327	*lacZ*	33±5	14±4
pTP-327-ArcA	P*arcA* + *lacZ*	ND^b^	236±25
pTP-327-Crp	P*crp* + *lacZ*	ND^b^	475±33
pTP-327-EtrA	P*etrA* + *lacZ*	ND^b^	199±21

a Absence/presence of 0.5 mM IPTG.

b No data.

Expression of *lacZ* from inserted promoters within pTP327-ArcA, pTP327-Crp, and pTP327-EtrA was at least 10 times above the background. The extent of elevation appeared to be promoter specific. These results suggest that pTP327 is able to fulfill the need as a reporter system in *S. oneidensis*. Note that this promoterless system should function not only in *S. oneidensis* but also any Gram-negative bacteria compatible with the broad-host *ori* within the plasmid.

### *In vivo* inter-regulation of ArcA, Crp, and EtrA

**Figure 4:**
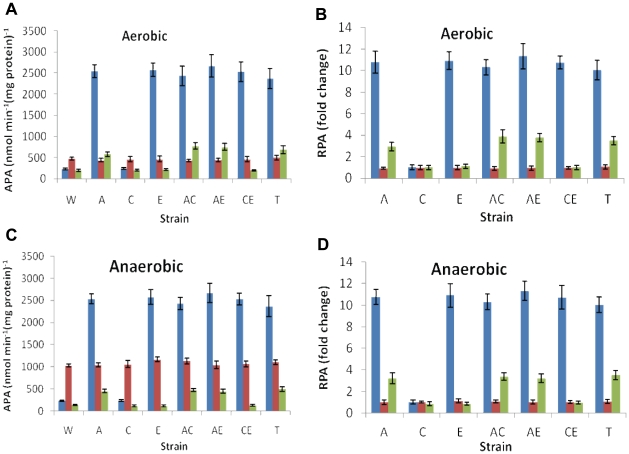
The *arcA* (blue), *crp* (red), and *etrA* (green) promoter activities in mutant strains under either aerobic (A, B) or anaerobic conditions (C, D). Promoter activities were determined by β-galactosidase activity measurements. Both absolute (A, C) and relative (B, D) activities are shown (refer the text for details). In all panels, W, MR-1; A, Δ*arcA*; C, Δ*crp*; E, Δ*etrA*; AC, Δ*arcA*Δ*crp*; AE, Δ*arcA*Δ*etrA*; CE, Δ*crp*Δ*etrA*; T, Δ*arcA*Δ*crp*Δ*etrA*.

The *in vivo* inter-regulation of *arcA*, *crp* and *etrA* gene transcription by ArcA, Crp, and EtrA was determined by comparing the expression pattern of the *arcA*, *crp*, or *etrA-lacZ* reporter constructs in *S. oneidensis* MR-1 with that in the mutant strains under aerobic, or anaerobic TMAO growth conditions. Since the background was far below the β-galactosidase activity obtained from plasmids containing inserted promoters, it was omitted in data analysis. Both the absolute β-galactosidase promoter activity (APA) of each promoter construct (Fig. 4A, 4C) and relative promoter activity (RPA) (Fig. 4B, 4D), which was calculated by normalizing APA of each mutant to APA of the wild type under the same condition were shown.

The promoter activity of the *S. oneidensis arcA* promoter in the wild type background was not strongly affected by growth conditions (Fig. 4A, 4C). This is somewhat unexpected because ArcA has been shown to be deeply involved in bacterial aerobiosis but not in anaerobic TMAO growth [13]. However, the deletion of either *arcA* or *etrA* resulted in a significant impact on the *arcA* promoter activity (Fig. 4B, 4D). The relative activities of the *arcA* promoter were elevated more than 10 times in the absence of the ArcA or EtrA gene products, suggesting that both proteins function as repressors. In contrast, the *arcA* promoter activity was not affected by the presence or absence of Crp under tested conditions. It is reasonable to assume that elevation of the β-galactosidase activities in *ΔarcAΔcrp* and *ΔcrpΔetrA* double mutants resulted from mutation in *arcA* and *etrA*, respectively. Interestingly, the *arcA* promoter activity was not further significantly increased in the *ΔarcAΔetrA* double or the triple mutant. These results may indicate an epistasis relationship between ArcA and EtrA.

The reporter results with the *crp* promoter are completely different from those observed with the *arcA* promoter. In this case, the transcription activity of the *crp* promoter was hardly affected by any deletion mutation (Fig. 4B, 4D). However, the presence of oxygen in the culture resulted in an approximately 2.5-fold decrease in the *crp* promoter activity, consistent with the idea that the Crp protein has a larger role in anaerobiosis than aerobiosis in *S. oneidensis* [11]. Based on the observation that no change in growth phenotype resulted from an *etrA* mutation and EtrA was present in the wild-type under aerobic and anaerobic conditions as revealed by proteomics (unpublished results), thus a constant activity from the *etrA* promoter was expected under tested conditions. Indeed, transcriptional activity of the *etrA* promoter was not sensitive to growth conditions. Surprisingly, however, the *arcA* deletion strains exhibited a substantial increase in *etrA* promoter activity, suggesting ArcA represses *etrA* transcript. On the contrary, *etrA* transcription was not affected by the *etrA* or *crp* deletions under the conditions tested (Fig. 4B, 4D).

It is apparent that the β-galactosidase reporter experiments for these promoters revealed some interesting findings. The *crp* gene is the most transcriptionally active with β-galactosidase levels at least two times higher than those of *arcA* and *etrA* (Fig. 4A, 4C). It is also the only gene whose expression is significantly influenced by oxygen but not by deletions of the major global regulator proteins, including Crp itself. Although the *etrA* gene was transcribed at the lowest level, its product appeared to be functional as evidenced by its role in the repression on the *arcA* promoter. Both *arcA* and *etrA* promoters were repressed by ArcA, but availability of oxygen hardly influenced transcription of these two genes.

### *In silico* analysis on interaction between ArcA, Crp, and EtrA and their promoters

**Figure 5:**
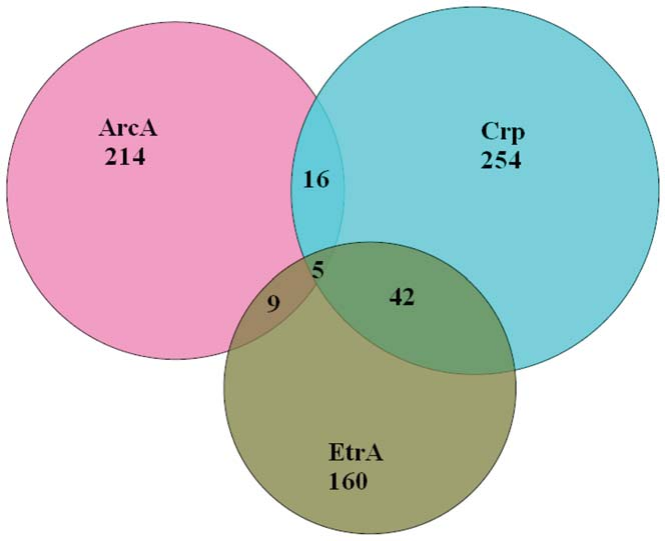
Venn diagram showing the number of *S. oneidensis* genes whose upstream regions contains ArcA-, Crp-, or EtrA-binding motifs.

The experimental data presented thus far have established the interplay between ArcA, Crp and EtrA in regulation of respiration of *S. oneidensis*. To further investigate whether such interplay occurs directly or indirectly, an *in silico* analysis was employed. Previously, we developed ArcA-binding weight matrices using sequences containing an ArcA binding motif derived from transcriptional profiling and EMSA [13], [26]. Genome scanning with the matrices revealed 209 operons whose upstream regions contain predicted ArcA binding motifs [13]. Here, we intended to identify the Crp- and EtrA-binding sites in the genome of *S. oneidensis* with the same strategy but different programs as described in Methods. The analysis with Regulatory Sequence Analysis Tools (RSAT) revealed 214, 254, and 160 genes containing predicted ArcA-, Crp-, and EtrA-binding motifs respectively (Fig. 5 and Table S2, S3, and S4 in Supporting Information). Interestingly, less than a dozen of genes were found to be likely under direct control of ArcA and EtrA. Among the 254 predicted Crp-binding motifs, none of these sites was located within upstream regions of *arcA*, *crp*, or *etrA*, consistent with the observation that expression of these genes were not altered in the *Δcrp* strain. On the contrary, an EtrA-binding motif was identified within the upstream region of *crp* while the promoter region of *arcA* contains a binding motif of its own.

### ArcA levels in *S. oneidensis*

**Figure 6:**
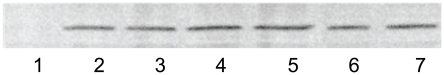
ArcA protein levels in various strains by quantitative immunoblotting assay. Lane 1, Δ*arcA* under aerobic conditions; 2, MR-1 under aerobic conditions; 3, MR-1 under anaerobic conditions; 4, Δ*etrA* under aerobic conditions; 5, Δ*etrA* under anaerobic conditions; 6, Δ*crp* under aerobic conditions; and 7, Δ*crp* under anaerobic conditions.

Although regulation of gene expression at the transcriptional level is indisputably the most important control in bacteria, different post-transcriptional regulation mechanisms have been identified and reported to be crucial in many biological processes [27]. To confirm the promoter activities obtained from the β-galactosidase assay and to investigate whether post-transcriptional control has a significant role in production of ArcA, Crp and EtrA, quantitative immunoblotting analysis was performed to examine the quantity of ArcA protein in MR-1, *Δcrp*, and *ΔetrA* cells under the same conditions as the β-galactosidase activity assay described above. The *ΔarcA* strain was included as the negative control. The amount of ArcA protein present in these sampled cells was measured as described in Methods. To ensure that signals obtained from the sampled cells were in the linear range, serial dilutions of whole-cell lysates and purified ArcA protein were tested (data not shown). The results indicate that ArcA levels are virtually identical in the wild type and *Δcrp* strains under either aerobic or anaerobic conditions, consistent with the observations from the β-galactosidase reporter analysis (Fig. 6). However, the amount of ArcA proteins increased only 2.3±0.7 and 1.9±0.5 times in the *ΔetrA* strain under aerobic and anaerobic conditions, respectively (Fig. 6). Compared to those obtained from the β -galactosidase activity assay, these increases were 3–5 times smaller. An explanation for this difference is that there were 5–7 copies of the plasmid for the transcriptional analysis and only one chromosomal copy for the protein immunoblotting assay.

## Discussion

*S. oneidensis* MR-1 is an intriguing microorganism in terms of its metabolic pathways. Its genome contains genes encoding many enzymes of mixed acid fermentation but it is not able to ferment [8], [28]. Thus the bacterium can only grow by means of aerobic and anaerobic respiration, in which it is renowned for its versatility. However, the mechanism by which *S. oneidensis* controls the transition from aerobiosis to anaerobiosis remains elusive despite several investigations since the predicted major player, EtrA, was identified 16 years ago [14]. It has become apparent that the function of major global regulators, such as ArcA, Crp, and EtrA, is altered in *S. oneidensis* compared to *E. coli* [9], [11], [13], [21]. Recently, such an alteration has been clearly demonstrated in regulation of arsenate respiration pathway in *Shewanella* sp. strain ANA-3 [29]. Crp, rather than EtrA or ArcA, is an essential regulator for arsenate respiration.

We previously argued that the major role of ArcA in *S. oneidensis* resides in aerobiosis [13]. This argument gains support from the significant impact of ArcA on *S. oneidensis* carbon utilization observed in this study. Interestingly, expression of *arcA* was hardly influenced by the availability of oxygen at either the transcriptional or protein product levels. This is generally in agreement with previous findings in a series of transcriptomic and proteomic profilings where expression of *arcA* was found to be insensitive to various treatments, such as an array of stresses [30]–[34] and up to 10 different electron acceptors under anaerobic conditions [35]. One exception was observed in proteomic profiling of Fe_2_O_3_-treated *S. oneidensis* MR-1 cells [36], which, however, contradicts the findings from another profiling study [35]. Given that phosphorylation is essential to *S. oneidensis* ArcA for its binding to DNA targets [13], it appears that the phosphorylation state rather than the amount of ArcA protein determines its regulatory functions.

The status of Crp as an important component of the respiration transcription response was reinforced in this study by the finding that Crp has a role in aerobic respiration as evidenced by its impact on carbon utilization. Although the underlying mechanism for this observation is currently unknown, it is unlikely that *S. oneidensis* Crp could function as the primary transcriptional regulator of carbon metabolism like its canonical counterpart in *E. coli* [37]. *S. oneidensis* is notoriously limited in its capability for utilizing carbon resources, especially six-carbon sugars [8]. Whether this phenomenon is related to a shift of Crp function is an interesting question. In addition, the involvement of Crp in aerobiosis was indicated by a significant reduction in cytochrome *c* synthesis in the *crp* deletion mutant in the presence of oxygen. The involvement of Crp in aerobiosis was further confirmed by a relatively high level of *crp* expression (compared to *arcA* and *etrA*) under aerobic conditions and the finding that Crp is among the most abundant regulatory proteins detected in a proteomic study [7].

In this study, a LacZ-reporter system for *S. oneidensis* was constructed and used to assess the promoter activities of *arcA*, *crp*, and *etrA*. The activity of the introduced promoter may be higher than its actual level because of multiple copies of the plasmid. Nevertheless, the system is functional and convenient in operation, and more importantly, it is expected to work with other Gram-negative bacteria which lack a *lacZ* analog in the genome. Using the system, an attempt to reveal the interactive control among these three major regulators in *S. oneidensis* was made. One of the most striking findings is that expression of *arcA* was negatively controlled by EtrA given that EtrA has no significant role in regulating anaerobic respiration of *S. oneidensis* [9]–[10]. Interestingly, both EtrA and ArcA repress *arcA* expression to a similar degree but removal of EtrA does not elicit an obvious phenotype, suggesting that the presence and phosphorylation state of ArcA rather than its net amount is more important for regulation in *S. oneidensis*.

The data from the promoter activity assay gain supports from the bioinformatics analysis in general. Independent expression of *arcA*, *crp*, or *etrA* from Crp is most likely due to the lack of Crp-binding motifs within upstream regions of these genes although other mechanisms such as activation (as in the canonical system) may have a role [37]. By identifying an ArcA-binding motif in close proximity to *arcA*, the *in silico* analysis reinforces that ArcA represses its own expression through a direct-control mechanism. On the contrary, repression of *arcA* expression by EtrA may possibly be indirect. It is worth noting that more than 60% of top 500 genes under control of either ArcA or Fnr have been shown to be mediated by both regulators directly or indirectly in *E. coli*, suggesting a great deal of functional overlap [38]. In *S. oneidensis*, genes (42) under direct control of both Crp and EtrA substantially exceed those (9) regulated by ArcA and EtrA (Fig. 5), implicating that Crp and EtrA may be more functionally related. These discrepancies suggest that mechanisms of cellular regulation concluded from *E. coli* may not reflect a general model for bacteria, even not within the same class of γ–proteobacteria.

The transcriptional regulatory network in *E. coli* has been extensively studied in recent years and many invaluable insights into this complex process have been obtained [23], [39]–[41]. Although understanding is still far from complete, the finding benefits numerous studies in other microorganisms. However, the results reported previously and presented here demonstrated that *S. oneidensis* differs from *E. coli* profoundly, especially in terms of the function of global regulators. We argue that the differences will lead to a *S. oneidensis* regulatory network which has little in common with the one defined in *E. coli*. To this point, this study is particularly of importance by providing useful information for understanding the regulatory network in *S. oneidensis*.

## Methods

### Bacterial strains, plasmids, and culture conditions

A list of all bacterial strains and plasmids used in this study is given in Table 1. *E. coli* and *S. oneidensis* strains under aerobic conditions were grown in Luria-Bertani (LB, Difco, Detroit, MI) medium at 37°C and at room temperature for genetic manipulations, respectively. When needed, the growth medium was supplemented with antibiotics at the following concentrations: ampicillin at 50 µg/ml, gentamycin at 15 µg/ml, and tetracycline at 15 µg/ml.

### Construction of deletion mutants

A series of deletion strains were constructed for this study. The *arcA* deletion mutant was generated and validated previously and used as the parental strain for constructing subsequent double and triple mutants [13]. Primers used for generating PCR products for mutagenesis are listed in Table S1 in the supplemental material. The mutagenesis process for construction of the *crp* and *etrA* single deletion mutants, named JZ0624 (*Δcrp*) and JZ2356 (*ΔetrA*) respectively, followed the procedure described elsewhere [42]. Double mutants JZ3988K-0624 (*ΔarcAΔcrp*), JZ3988K-2356 (*ΔarcAΔetrA*), and JZ0624-2356 (*ΔcrpΔetrA*) were generated by repeating the mutagenesis procedure by introduction of a plasmid containing the latter gene deletion structure into the former gene deletion strain. The triple mutant JZ3988K-0524-2356 (*ΔarcAΔcrpΔetrA*) was also constructed in this way by introducing the *etrA* deletion structure into the *ΔarcAΔcrp* double mutant. The deletion(s) in each strain were verified by PCR and DNA sequencing.

### Physiological characterization of the mutant strains

M1 defined medium containing 0.02% (w/v) of vitamin-free Casamino Acids and 30 mM lactate was named as M1-L and used in all physiological experiments [43]. Growth of the mutant strains under aerobic or anaerobic conditions was determined by recording growth curves in triplicate with a Bioscreen C microbiology reader (Labsystems Oy, Helsinki, Finland) with the wild-type as the control as described previously [13]. For anaerobic growth, exponential phase cultures grown under aerobic conditions were centrifuged, purged in nitrogen and suspended in fresh medium to approximately ∼1×10^5^ cells/ml in an anaerobic glove box. Electron acceptors tested in this study included fumarate (20 mM), nitrate (2 mM), nitrite (1 mM), thiosulfate (3 mM), TMAO (20 mM), and DMSO (20 mM). For electron acceptors containing metals including MnO_2_ (5 mM), ferric citrate (10 mM), and cobalt(III)-EDTA (200 µM), growth was monitored by the color change of the cultures and cell counting under a microscope (Nikon Optiphot, Nikon, Japan).

### Biolog phenotype microarray screen

Phenotype microarray (PM) plates (Biolog Inc., Hayward, California) were used to examine carbon utilization of *S. oneidensis* MR-1 and the deletion mutant strains in duplicate. Cells of MR-1 and each mutant strain were prepared under aerobic conditions according to the manufacturer's instructions. Incubation and recording of phenotypic data were performed with an OmniLog instrument. The altered phenotypes of the mutants were assessed by comparison to the parental strain MR-1 with the OmniLog PM bioinformatics software.

### Biochemical methods

All mutant strains and *S. oneidensis* MR-1 were grown to the late exponential phase either in M1-L under aerobic conditions (OD_600_≈0.6) or in M1-L supplemented with 20 mM TMAO under anaerobic conditions (OD_600_≈0.25). The cells were harvested and then were lysed with lysis buffer (0.25 M Tris/HCl, (pH 7.5), 0.5% Trion-X100). Protein concentration was determined with a bicinchoninic acid assay kit with bovine serum albumin (BSA) as a standard according to the manufacturer's instructions (Pierce Chemical). The amount of heme *c* was assessed following the procedure described elsewhere [11], [44].

### Development of an *S. oneidensis* lacZ reporter system

A *lacZ* reporter system for *S. oneidensis* was developed in this study. The *E. coli lacZ* gene was obtained by PCR amplification with pBlueSTAR-1 (Novagen) as the template using primers LacZ-F/R listed in Table S1. The approximately 3 kb PCR product was inserted into the *Kpn*I site of the *lacZα* gene within the broad-host plasmid pCM62 [18], resulting in pTP325. A short synthetic DNA fragment generated by primers Linker-F/R (Table S1) was used to replace the *Plac* promoter between *Ase*I and *Hind*III sites within pTP325. This final plasmid containing promoterless *E. coli lacZ* gene was designated as pTP327.

To construct the *arcA-lacZ*, *crp-lacZ*, and *etrA-lacZ* reporters, the *arcA*, *crp*, and *etrA* promoter DNA fragments were first generated by PCR with primers SO3988-PF/R, SO0624-PF/PR, and SO2356-PF/PR listed in Table S1. These PCR products were then inserted into the *Xho*I and *Hind*III restriction sites of pTP327 individually, resulting in pTP327-3988, pTP327-0624, and pTP327-2356, respectively. After verification by DNA sequencing, the reporter plasmids were moved into each *S. oneidensis* mutant strain used in this study and MR-1 by conjugation. The resulting strains (Table 1) were maintained in LB (aerobic) or LB supplemented with 20 mM lactate, 20 mM TMAO (anaerobic) containing 15 µg/ml tetracycline.

### β-Galactosidase activity assay

This assay was performed using the High Sensitivity β-Galactosidase Assay Kit from Stratagene. The bacterial cells, collected from aerobic log phase (30°C, OD_600_ = 0.3∼0.4), anaerobic TMAO (room temperature for 14.5 hr) growth conditions, were harvested by centrifugation, washed with PBS (phosphate buffered saline), and treated with lysis buffer (0.25 M Tris/HCl, (pH 7.5), 0.5% Trion-X100). The resulting soluble protein was collected after centrifugation to remove the insoluble cellular fractions, and subjected to the enzyme assay according to manufacturer's instructions. The β-galactosidase activity was determined by monitoring color development at 575 nm every minute for 30 min by using a Synergy 2 Multi-Detection Microplate Reader. The protein concentration of the cell lysates was determined using a Bradford assay with BSA as a standard.

### Quantitative Immunoblotting assays for ArcA

Expression and purification of recombinant *S. oneidensis* His-tagged ArcA protein was performed as described previously [13]. Rabbit polyclonal antibodies against the recombinant ArcA were prepared in accordance with standard protocols provided by the manufacturer (Lampire Biological Laboratories, Pipersville, Pa.) and used for immunoblotting analysis.

The cells collected for the β-galactosidase activity assay were used for quantitative immunoblotting assays. For these experiments, cell samples were thawed, washed once with TE buffer (10 mM Tris [pH 8.0], 1 mM EDTA), and resuspended to an optical density at 600 nm (OD_600_) of 1.0 in lysis buffer (50 mM Tris [pH 8.0], 1 mM EDTA, 100 mM NaCl). The total protein concentration of the cell lysates was then determined by the bicinchoninic acid assay (Pierce Chemical). Samples were loaded onto SDS-12% polyacrylamide gels and either stained with Coomassie brilliant blue or electrophoretically transferred to nitrocellulose according to manufacturer's instructions (Bio-Rad).The gels were blotted for 1 h at 50 V using a Criterion blotter (Bio-Rad). An appropriate amount of supernatant was chosen by trial and error for immunoblotting analysis to ensure that the signals observed were not saturated. Images were visualized and quantified with the FluorChem Imaging System in conjunction with AlphaEaseFC software. The linear range for the signal was established by serial dilutions of whole-cell lysates and purified ArcA protein.

### CRP and EtrA binding motif analysis

*E. coli* operons under direct control of CRP and EtrA were derived from reports published previously [45]–[47]. The promoter regions of obtained operons were subjected to screening for common binding motifs, which was subsequently transformed to a weight matrix using AlignACE [48]. The whole genome was then scanned for putative binding motifs with the weight matrix using RSAT with the default setting [49].

## Supporting Information

Table S1 Primers used in this study.
(DOC)

Table S2 Predicted ArcA binding sites in the genome of *S. oneidensis*.
(XLS)

Table S3 Predicted Crp binding sites in the genome of *S. oneidensis*.
(XLS)

Table S4 Predicted EtrA binding sites in the genome of *S. oneidensis*.
(XLS)
