# Supplementary material for: Physiological Roles of ArcA, Crp, and EtrA and Their Interactive Control on Aerobic and Anaerobic Respiration in Shewanella oneidensis
Source: PLoS One. 2010 Dec 28;5(12):e15295. doi: 10.1371/journal.pone.0015295 (PMC3011009; doi:10.1371/journal.pone.0015295)
Supplement: Table S1 — Primers used in this study. (DOC) [file pone.0015295.s001.doc]

| **TABLE S1. Primers used in this study** | |
| --- | --- |
| Mutagenesis |  |
| SO0624-5-F | GCCTGTATTTCACCTGGT |
| SO0624-5-R | TGTTTAAACTTAGTGGATGGGGGTCTGGTTTTGGCTTACC |
| SO0624-3-F | CCCATCCACTAAGTTTAAACATGGTTGCTCCCGCGAAACTGTG |
| SO0624-3-R | CATGTTTTCGACATTCGCTCG |
| SO2356-5-F | ACTTAGCCCATCAACTCACC |
| SO2356-5-R | TGTTTAAACTTAGTGGATGGGATTCTGCTCTATTGTCATGTCG |
| SO2356-3-F | CCCATCCACTAAGTTTAAACAAACCATTAGCCGCTTACTCG |
| SO2356-3-R | TACGCATTAAATGCCAATCG |
|  |  |
| Reporter system |  |
| LacZ-F | GCAAGGTACCGAGCTCGAATTCACTGGCCGTCGTTTTACAACGTC |
| LacZ-R | GTTAGGTACCTTATTTTTGACACCAGACCAACTGGTAATGGTAGC |
| Linker-F | TAATACCGGTCCATGGAGTACTCTCGAGATGACCATGATTACGCCA |
| Linker-R | TCGAACCGCATTAGTACCAGTAGAGCTCTCATGAGGTACCTGGCCATA |
| SO0624-PF | aactcgagTGTGCAACAGAACTTAATCC |
| SO0624-PR | TGGTAGATAAGCTTGGCGTAATCATGGTCATGTCGATGTTCCTCGATTG |
| SO2356-PF | acctcgagGGCGCTTTAGTCGGCGGCT |
| SO2356-PR | TGGTAGATAAGCTTGGCGTAATCATGGTCATGTTAATCCACTGCAGCCATG |
| SO3988-PF | aactcgagGCGTGAACTCGCTCACAAG |
| SO3988-PR | TGGTAGATAAGCTTGGCGTAATCATGGTCATATTAAGTACCTAAATTTTAACC |
